# Heat Release Property and Fire Performance of the Nomex/Cotton Blend Fabric Treated with a Nonformaldehyde Organophosphorus System

**DOI:** 10.3390/polym8090327

**Published:** 2016-09-02

**Authors:** Charles Q. Yang, Qin Chen

**Affiliations:** 1 Department of Textiles, Merchandising and Interiors, the University of Georgia, Athens, GA 30602, USA; chen.qin25@uga.edu; 2 College of Chemistry, Chemical Engineering & Biotechnology, Donghua University, Shanghai 201620, China

**Keywords:** cotton, Nomex^®^, Nomex^®^/cotton blend fabrics, flame retardant finishing, flame retardant textiles, fire-resistant protective clothing

## Abstract

Blending Nomex^®^ with cotton improves its affordability and serviceability. Because cotton is a highly flammable fiber, Nomex^®^/cotton blend fabrics containing more than 20% cotton require flame-retardant treatment. In this research, combination of a hydroxyl functional organophosphorus oligmer (HFPO) and 1,2,3,4-butanetetracarboxylic acid (BTCA) was used for flame retardant finishing of the 65/35 Nomex^®^/cotton blend woven fabric. The system contains HFPO as a flame retardant, BTCA as a bonding agent, and triethenolamine (TEA) as a reactive additive used to enhance the performance of HFPO/BTCA. Addition of TEA improves the hydrolysis resistance of the HFPO/BTCA crosslinked polymeric network on the blend fabric. Additionally, TEA enhances HFPO’s flame retardant performance by reducing formation of calcium salts and also by providing synergistic nitrogen to the treated blend fabric. The Nomex^®^/cotton blend fabric treated with the HFPO/BTCA/TEA system shows high flame resistance and high laundering durability at a relatively low HFPO concentration of 8% (*w*/*w*). The heat release properties of the treated Nomex^®^/cotton blend fabric were measured using microscale combustion calorimetry. The functions of BTCA; HFPO and TEA on the Nomex^®^/cotton blend fabric were elucidated based on the heat release properties, char formation, and fire performance of the treated blend fabric.

## 1. Introduction

Nomex^®^, a poly(meta-aramide) fiber, was developed commercially as a heat resistant synthetic fiber in the 1960s. Nomex^®^ was specified for use as protective clothing for aerospace personnel by NASA in 1971 [1]. The chemistry and applications of Nomex^®^ were thoroughly reviewed elsewhere [2,3]. Nomex^®^ has been a widely used flame retardant fiber since 1960s, and today it is still one of the most successful high performance technical fibers in the industry [4,5,6]. However, high cost and low comfortability of Nomex^®^ have limited its wider applications. Blending Nomex^®^ with cotton reduces cost but also improves comfortability of the fabrics. Because cotton is a highly flammable fiber, Nomex^®^/cotton blend fabrics containing more than 20% cotton are not self-extinguishable [7,8]. A flame-retardant finishing procedure becomes necessary to make such blend fabrics fire-resistant.

**Scheme 1:**
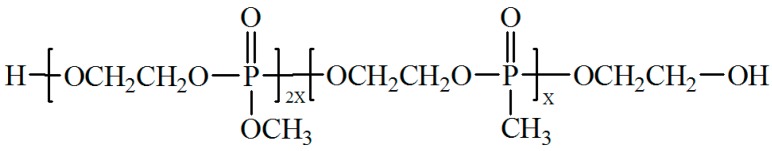
Hydroxy-functional organophosphorus oligomer (HFPO).

Previously, the commercially available hydroxy-functional organophosphorus oligomer (HFPO) shown in Scheme 1 was used as a flame retardant finishing agent for cotton. Because HFPO does not have a functional group reactive to cotton, it is necessary to use a bonding agent, such as dimethyloldihydroxyethyleneurea (DMDHEU) and trimethylolmelamine (TMM), so that HFPO can be covalently bound to cotton [9,10,11,12]. HFPO-based flame retardant systems were also applied to nylon/cotton blends used for protective clothing [13,14].

Since DMDHEU and TMM are formaldehyde-based reagents, formaldehyde emission would be inevitable during production, use and storage of those flame resistant garments. Formaldehyde was a known “probable carcinogen” in 1987 [15]. The risk of formaldehyde exposure was upgraded to “carcinogenic to humans” by WHO International Agency for Research on Cancer in 2004 [16]. Epidemiology studies showed that workers exposed to formaldehyde in their workplaces were at increased risk of leukemia and brain cancer compared with general public [17,18]. The proved link between cancer risk and exposure to formaldehyde emission in textile industry makes it imperative to develop formaldehyde-free alternative flame retardant systems.

The combination of HFPO and 1,2,3,4-butanetetracrboxylic acid (BTCA) was applied for flame retardant finishing of cotton and 65/35 Nomex^®^/cotton blend fabrics previously [19,20,21]. Triethenolamine (TEA) was used as an additive to improve the performance of the treated Nomex^®^/cotton fabric [21]. The current research was a continuation of the previous study on the flame retardant Nomex^®^/cotton fabric. Its focus was to elucidate the chemistry of the Nomex^®^/cotton blend fabric treated with HFPO, BTCA and TEA based heat release properties, char yield and fire performance of the treated Nomex^®^/cotton blend fabric.

## 2. Experimental

### 2.1. Materials

The 65/35 (*w*/*w*) Nomex^®^/cotton blend fabric was a twill weave military fabric with three-color woodland camouflage weighing 219 g/m^2^ produced in China. HFPO with the commercial name of “Fyroltex^®^ HP” (also known previously as “Fyrol^®^ 51”, CAS Registry No. 70715-06-9) was supplied by Supresta (formerly Akzo Nobel Phosphorus Chemical Division), Dobbs Ferry, NY, USA. BTCA, TEA and hypophosphorous acid (H_3_PO_2_) were all reagent-grade chemicals supplied by Aldrich, Milwaukee, WI, USA.

### 2.2. Fabric Treatment and Laundering Procedures

The fabric was first immersed in a solution containing HFPO, BTCA, the catalyst and TEA, passed through a laboratory padder with two dips and two nips, dried at 90 °C for 5 min and finally cured at a specified temperature. H_3_PO_2_ was used as the catalyst at 50% of BTCA (*w*/*w*). The fabric was also treated with BTCA and NaH_2_PO_2_ (catalyst) similarly without HFPO, and it was cured at 170 °C for 3 min. All concentrations presented here were based on weight (*w*/*w*, %). The wet pick-up of the padded Nomex^®^/cotton fabric was approximately 60% ± 2%. After curing, the treated fabric was subjected to a specified number of home laundering washing/drying cycles using a standard reference detergent (AATCC Detergent 1993) according to AATCC Test Method 124-1996. The water temperature for laundering was approximately 46 °C.

### 2.3. Evaluation of Flame Retardant Performance and Stiffness of the Fabric

The fabric vertical burning flammability was measured according to ASTM Standard Method D6413 (“standard test method for flame resistance of textiles (vertical test)”). The limiting oxygen index (LOI) of the fabrics was measured according to ASTM Standard Method D2863 (“standard test method for measuring the minimum oxygen concentration to support candle-like combustion of plastics (oxygen index)”). The fabric stiffness was measured according to ASTM Standard Method D6828 (“standard test method for stiffness of fabric by blade/slot procedure”) using a “Handle-O-Meter” tester (Model 211-300) manufactured by Thwing-Albert, Philadelphia, PA, USA. The slot width was 5 mm, and the beam size was 1000 g. The fabric stiffness data presented here were the mean of 5 measurements for each fabric sample.

### 2.4. Measurement of “Percent Fixation”

A fabric specimen was weighed: (1) before treatment (*W*_0_); (2) after treatment and before washing (*W*_1_); and (3) after treatment and subsequent washing (*W*_2_). All specimens were weighed after being conditioned for 24 h. A fabric’s percent fixation was calculated by Equation (1). “Fixation %” represents the weight percentage of the applied HFPO/BTCA/TEA chemically bound to the fabric substrate with respect to the original prewash value.

Fixation % = (*W*_2_ − *W*_0_)/(*W*_1_ − *W*_0_) × 100% (1)

### 2.5. Determination of Phosphorus and Calcium Concentration on a Treated Fabric

Approximately 2 g of a fabric sample taken from three different areas in a fabric specimen (10 inch × 12 inch) were ground in a Wiley mill into a powder to ensure sample uniformity. Two milliliters of concentrated H_2_SO_4_ was added to 0.1 g powder sample in a beaker. Ten milliliters of 30% H_2_O_2_ were added dropwise to the mixture, allowing the reactions to subside between drops. The reaction mixture was heated at approximately 250 °C to digest the powder and to evaporate the water until dense SO_3_ vapor was produced. The completely digested fabric sample became a clear solution. It was transferred to a 50 mL volumetric flask, and then diluted with deionized water to the mark. The sample thus prepared was analyzed with a Thermo-Farrell-Ash Model 965 inductively coupled plasma atomic emission spectrometer (Thermal-Farrel-Ash Corporation, Franklin, MA, USA) to determine the phosphorus concentration. The percent phosphorus retention was calculated by the following equation: (the phosphorus concentration of the fabric after laundering) ÷ (that before laundering) × 100%.

### 2.6. Microscale Combustion Calorimetry (MCC) Measurement

The MCC measurements of the fabric samples were conducted using a microscale combustion calorimeter (model “MCC-2”) produced by Govmark, Farmingdale, NY, USA, according to ASTM D7309 (Method A) (“standard test method for determining flammability characteristics of plastics and other solid materials using microscale combustion calorimetry”). A fabric sample was first ground in a Wiley mill into a homogeneous powder. A powder sample thus prepared (∼5 mg) was loaded to the instrument and then heated to a specified temperature using a linear heating rate (1 °C/s) in a stream of nitrogen flowing at 80 cm^3^/min. The thermal degradation products of the sample in nitrogen were mixed with a 20 cm^3^/min stream of oxygen prior to entering the 900 °C combustion furnace. Each sample was run in three replicates. The MCC parameters of a specimen presented in this paper were the mean of three measurements.

## 3. Results and Discussion

### 3.1. Treatment of the Nomex^®^/Cotton Fabric by BTCA

Polycarboxylic acids exemplified by BTCA have long been used as crosslinking agents for cotton and wood cellulose [22,23]. They were known as formaldehyde-free wrinkle resistant finishing agents for cotton. Polycarboxylic acids were first reported as flame retardants for cotton carpets in 2002 [24]. In our previous research, we investigated the applications of different polycarboxylic acids, such as BTCA, citric acid and succinic acid, to flame retardant finishing of cotton fleece [25,26,27,28]. It was discovered that BTCA and other polycarboxylic acids were able to reduce the flammability of cotton fleece from “Class 3” (high flammability) to “Class 1” (normal flammability) according to the U.S. government regulation 16 CFR 1610.

**Figure 1:**
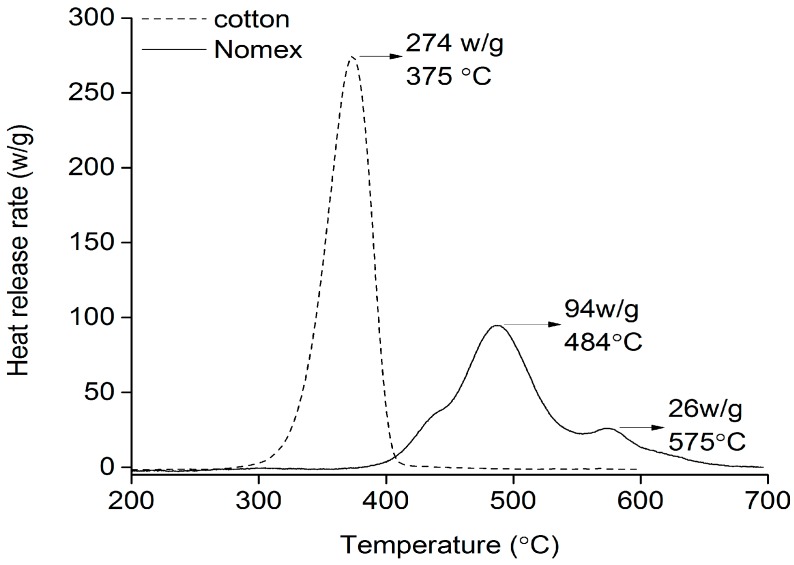
The heat release rate vs. temperature curves of cotton and Nomex^®^ fabrics.

**Figure 2:**
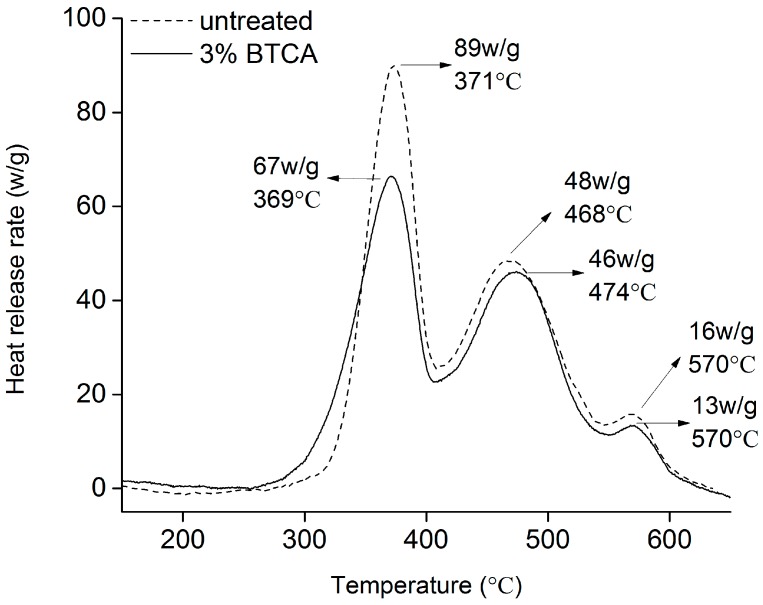
The heat release rate vs. temperature curves of the untreated Nomex^®^/cotton fabric and that treated with 3% 1,2,3,4-butanetetracarboxylic acid (BTCA), cured at 170 °C for 3 min and finally subjected to one home laundering cycle.

In this research, the effects of BTCA on the peak heat release rate (PHRR), heat release capacity (HRC), percent char yield and vertical burning flammability of cotton, Nomex^®^ and the 65/35 Nomex^®^/cotton blend fabrics are evaluated. The PHRR of 100% cotton is 274 w/g (Figure 1), and it becomes 89 w/g for the cotton in the Nomex^®^/cotton blend (Figure 2) because the blend contains approximately 35% cotton. The PHRR of cotton in the Nomex^®^/cotton blend treated with 3% BTCA is 67 w/g, representing 25% reduction, whereas the temperature at PHRR (*T*_PHRR_) changes very little (Figure 2). The BTCA treatment has no effect on both PHRR and *T*_PHRR_ of Nomex^®^ in the blend, as shown in Figure 2.

Presented in Table 1 is the PHRR, HRC and char yield of cotton on the Nomex^®^/cotton blend treated with BTCA with different concentrations. The decrease in PHRR and HRC of cotton was in the ranges of 21%–25% and 19%–22%, respectively, which appears to be independent of BTCA concentration. The char yield of the untreated blend fabric was 31.2%, which is mostly from the decomposition of Nomex^®^ in the blend. The data show that the BTCA treatment caused little increase in char yield (Table 1). The LOI of the treated blend fabric becomes marginally higher than that of the untreated blend fabric. All the fabric samples fail the vertical flammability test (Table 2).

### 3.2. Treatment of the Nomex^®^/Cotton Fabric by BTCA and HFPO

**Scheme 2:**
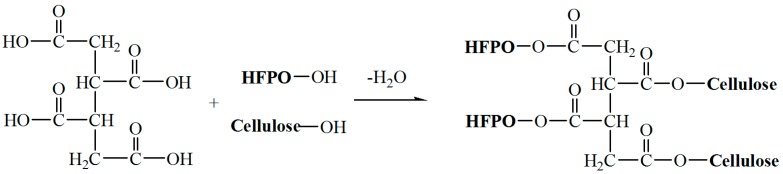
Esterification of BTCA by HFPO and cotton cellulose.

**Figure 3:**
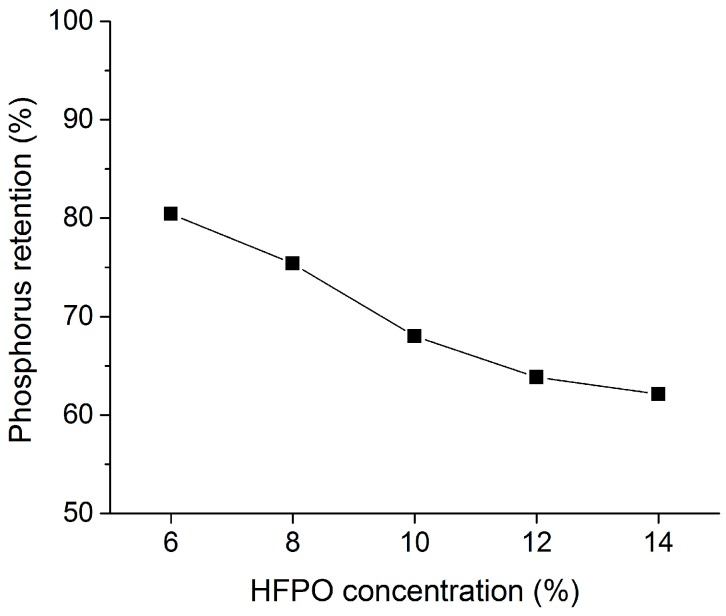
The phosphorus retention of the Nomex^®^/cotton fabric treated with 3% BTCA and HFPO with different concentrations, cured at 170 °C for 3 min and finally subjected to one home laundering cycle.

The 65/35 Nomex^®^/cotton blend fabric is treated with 3% BTCA in combination with HFPO at concentration ranging from 6% to 14%, cured at 170 °C for 3 min and finally subjected to one home washing cycle. Presented in Table 3 is the phosphorus concentration of the fabric thus treated before and after the washing procedure. Shown in Figure 3 is the percent phosphorus retentions, which is the percentage of the applied phosphorus bound to the blend fabric with respect to their prewash values. The data demonstrate that as the HFPO concentration is increased from 6% to 14%, the phosphorus concentration on the fabric after wash increases from 0.37% to 0.64% (Table 3) whereas the percent phosphorus retention decreases from 80% to 62%, respectively (Figure 3). Both HFPO and cotton cellulose are hydroxy-functional compounds. They compete to esterify BTCA on the fabric as shown in Scheme 2. At a constant BTCA concentration, increasing HFPO concentration causes the esterification of HFPO/BTCA to increase and consequently esterification of cotton/BTCA to decrease. As a result, percent of the HFPO bound to cotton decreases in spite of the increase in total HFPO bound to cotton as shown in Figure 3 and Table 3, respectively. This is because the bonding of HFPO to cotton requires BTCA to esterify both cotton and HFPO. Therefore, increasing HFPO concentration increases the phosphorus bound to cotton due to an increase in HFPO/BTCA bonding, but it also reduces the percentage of HFPO bound to cotton because it reduces BTCA/cotton bonding. The data in Figure 3 show that 80% of the HFPO was bound to cotton at 6% HFPO. The phosphorus retention decreases to 64% at 12% HFPO. Increasing the HFPO concentration further to 14% reduces the phosphorus retention to 62%.

The calcium concentration of the treated blend fabric was also presented in Table 3. Calcium is an element having significant impact on the flame retarding performance of phosphorus-based flame retardants on cotton. Previously, we found that calcium was bound to the cotton treated with HFPO/BTCA after multiple launderings due to the formation of insoluble calcium salts [20]. Such calcium salts is detrimental to the flame retarding properties of phosphorus-based flame retardants on cotton and cotton blends [20,21]. In this research, calcium is detected on the Nomex/cotton blend fabric treated with HFPO/BTCA. Calcium concentration decreases from 0.075% to 0.065% as the HFPO concentration for the treatment is increased from 6% to 14% (Table 3). The declining calcium concentrations at higher HFPO concentrations shown in Table 3 is due to increasing esterification of BTCA/HFPO at higher HFPO concentration, which reduces the quantity of free carboxy of BTCA on cotton available for forming calcium salt.

**Figure 4:**
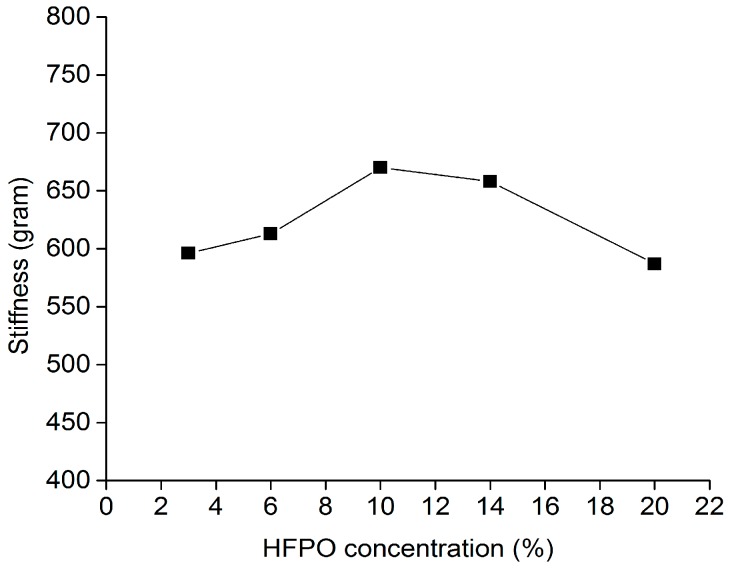
The stiffness of Nomex^®^/cotton fabric treated with 3% BTCA and HFPO at different concentration, cured at 170 °C for 3 min and finally subjected to one home laundering cycle.

Presented in Figure 4 is the stiffness of the blend fabric treated with the combination of 3% BTCA and HFPO at different concentrations, cured at 170 °C for 3 min and finally subjected to one washing cycle. Because HFPO has two hydroxyl groups in its molecule whereas BTCA has four carboxyl groups, it is most likely that the reactions between HFPO and BTCA form a crosslinked polymeric network on the Nomex^®^/cotton blend fabric. The fabric stiffness increases significantly when the HFPO concentration is increased to 10%–14% (Figure 4), which supports the hypothesis that the reactions of BTCA and HFPO forms crosslinked polymeric networks. Figure 4 also shows that formation of such crosslinked polymeric network is dependent on the HFPO/BTCA ratio. The same phenomenon was observed on the nylon/cotton blend fabrics treated with HFPO and DMDHEU in our previous research [29].

**Figure 5:**
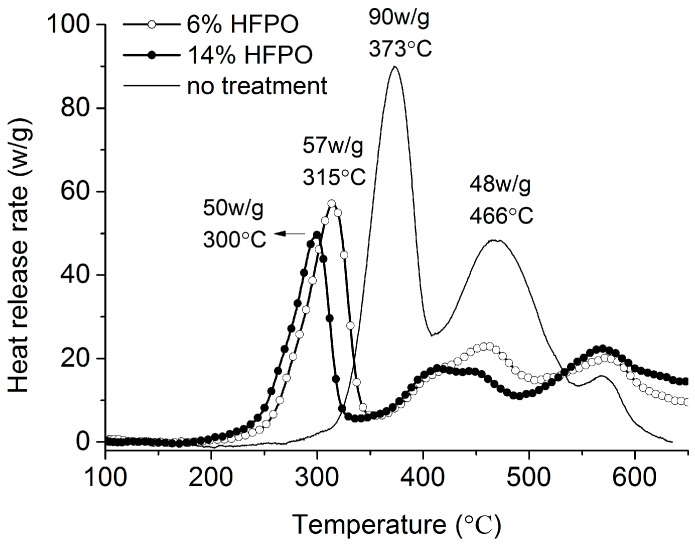
The heat release rate vs. temperature curves of the Nomex^®^/cotton fabric treated with 3% BTCA and: (1) no HFPO; (2) 6% HFPO; and (3) 14% HFPO, cured at 170 °C for 3 min and finally subjected to one home laundering cycle.

Presented in Table 4 are HRC, PHRR, total heat release (THR), temperature at PHRR (*T*_PHRR_) and char yield of the Nomex^®^/cotton blend fabric treated with 3% BTCA and HFPO at different concentrations. The HRR vs. temperature curves of the Nomex^®^/cotton blend without treatment and that treated with 3% BTCA and HFPO (6% and 14%) are shown in Figure 5. Drastic changes take place in the HRR vs. temperature curves when the blend fabric is treated with HFPO/BTCA. Without treatment, the HRR of cotton reaches its peak (90 w/g) at 371 °C. When the blend fabric is treated with 3% BTCA, the HRR peak (67 w/g) appears at 369 °C (Figure 2). When 6% HFPO is added to the treatment, the HRR peak decreases to 57 w/g at 315 °C (Figure 5). The char yield was 31.2% and 31.4% for the untreated fabric and that treated by 3% BTCA, respectively (Table 1). The char yield increases considerably to 41.9% when 6% HFPO is added for the treatment (Table 4). HRC, PHRR, total heat release (THR) and *T*_PHRR_ all decrease and char yield increases as the HFPO concentration is increased from 6% to 14% (Figure 5 and Table 4). The data also demonstrate that the changes in heat release properties and char yield are dependent on HFPO concentration (Table 4). Those significant changes are obviously due to the presence of phosphorus on the fabric, which changes cellulose degradation process to promote dehydration and also lowers the degradation temperature. Figure 5 also shows that the HFPO/BTCA system reduces the PHRR of poly(meta-aramide) on the blend.

The LOI and vertical burning flammability data of the Nomex^®^/cotton blend thus treated are presented in Table 5. Apparently, the HFPO/BTCA system is effective in imparting flame retarding properties to the blend fabric. The use of 6% HFPO in the flame retardant system drastically reduces the vertical burning char length from >300 mm (total burning) to 51 mm and increases LOI from 22.8% to 27.6%. The char length decreases and LOI increases significantly as the HFPO concentration increases from 6% to 14% (Table 5).

### 3.3. Treatment of the Nomex^®^/Cotton Fabric by BTCA, HFPO, and TEA

To study the chemical reactions of TEA with HFPO/BTCA, the fabric is treated with 12% HFPO, 3% BTCA and TEA with concentration ranging from 1% to 4%. Since TEA is a base, the traditional catalyst (NaH_2_PO_2_) is replaced by the acidic H_3_PO_2_. The final pH of all HFPO/BTCA/TEA solutions is adjusted to ~2.8 by adding NaOH or HCl depending on the quantity of TEA added to a solution. The fabric thus treated is cured at 170 °C for 3 min and finally subjected one regular home laundering cycle. To simulate a washing condition using water with high hardness, the fabric thus treated is subjected to a second washing cycle, in which 0.01% Ca(NO_3_)_2_ is added to the laundering solution to raise water hardness.

**Figure 6:**
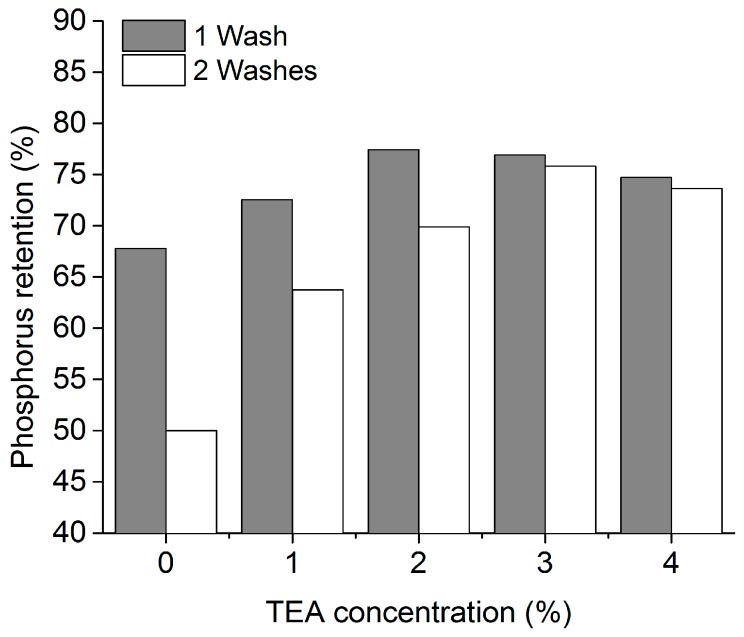
The phosphorus retention (%) of the Nomex^®^/cotton fabric treated with 12% HFPO, 3% BTCA and TEA with different concentrations, cured at 170 °C for 3 min and subjected to one regular wash followed by a second wash with 0.01% Ca(NO_3_)_2_ added.

The phosphorus concentrations before wash, after one wash and after two washes are shown as *P*_0_, *P*_1_ and *P*_2_, respectively, in Table 6. The phosphorus retentions (%) after one and two washes shown in Figure 6 are defined as (*P*_1_/*P*_0_) × 100% and (*P*_2_/*P*_0_) × 100%, respectively. Table 6 shows that the phosphorus concentration is in the vicinity of 0.91% for all the treated fabric samples before washing. When the treated fabric is subjected to a regular wash, phosphorus concentration (*P*_1_) on the treated fabric increases from 0.61 to the maximum (0.72) as the TEA concentration is raised from 0% to 2%. After the second washing procedure, the fabric treated without TEA retains 74% of the phosphorus after the first wash whereas that treated with 1% TEA retained 89% of the phosphorus after the first wash (Table 6). The fabric treated using 3% TEA has the highest phosphorus concentration of 0.69%, which reveals that the fabric retained 99% of the phosphorus after the first wash. When the TEA concentration is increased to 4%, the phosphorus concentration decreases to 0.67% (Table 6). Figure 6 shows that after the second wash, phosphorus retention for the fabric treated without TEA is 50.0%, whereas that for the fabric treated with 3% TEA was 75.8%. Further increasing TEA to 4% slightly reduces the phosphorus retention to 73.6%.

**Scheme 3:**
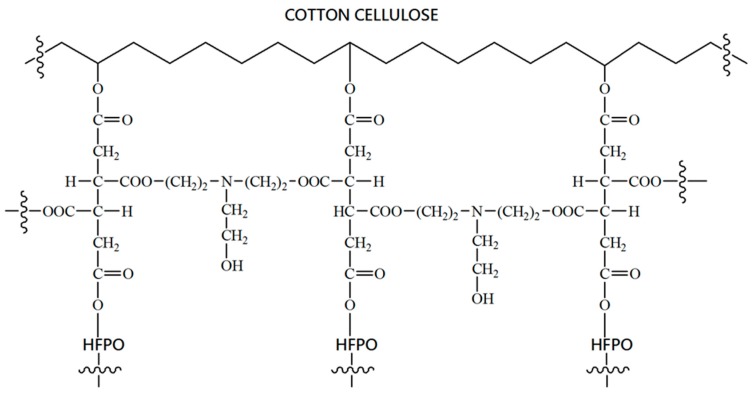
Formation of HFPO/BTCA/TEA/cotton crosslinked network.

The data presented here indicate that adding TEA as an additive to the HFPO/BTCA system significantly increases phosphorus retention of the treated fabric subjected to launderings. TEA has three hydroxy groups in its molecule and is able to esterify BTCA to form a HFPO/BTCA/TEA/cotton crosslinked network as shown in Scheme 3, thus improving hydrolysis-resistance of the HFPO bound onto the blend. The data also demonstrate that the TEA concentration has an optimum range (~3%). Since TEA, HFPO and cotton are all hydroxy-functional compounds, a further increase in TEA concentration reduces the esterification between cotton and BTCA and that between HFPO and BTCA, thus reducing the bonding of HFPO to cotton and lowering the phosphorus retention.

**Figure 7:**
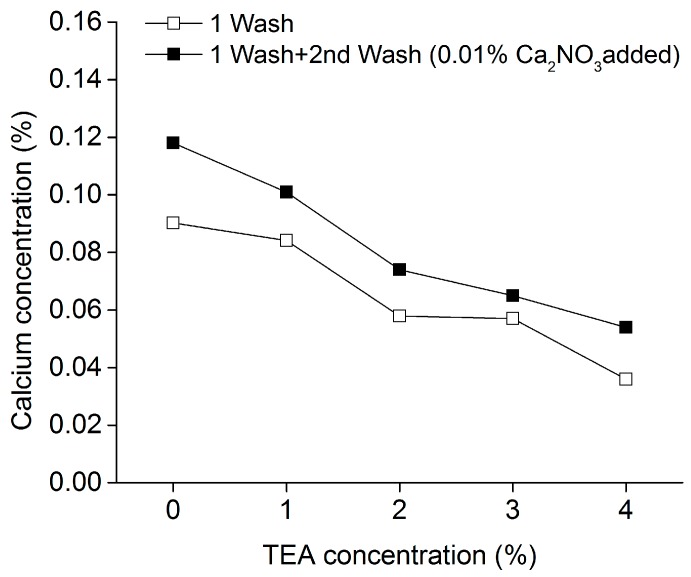
The calcium concentration (%) of the Nomex^®^/cotton fabric treated with 12% HFPO, 3% BTCA and TEA with different concentrations, cured at 170 °C for 3 min and subjected to one regular wash followed by a second wash with 0.01% Ca(NO_3_)_2_ added.

Presented in Figure 7 are the calcium concentrations of the Nomex^®^/cotton blend fabric treated with 12.0% HFPO, 3.0% BTCA, and TEA at concentration ranging from 1% to 4% and subjected to two washing cycles against TEA concentration. The calcium concentration on the treated fabric after the first washing cycles decreases significantly as the TEA concentration is increased from 0% to 4% (Figure 7). Esterification of BTCA by TEA reduces the concentration of free carboxy group on the fabric and consequently reduces formation of calcium salt. Adding Ca(NO_3_)_2_ in the second washing procedure leads to more calcium salt formation of the fabric. Consequently, TEA shows a more profound effect on the reduction of the calcium salt on the fabric (Figure 7). The calcium concentration of the fabric treated with HFPO/BTCA without TEA is 0.118% after the second wash, whereas it drastically decreases to 0.054% when 4% TEA is used (Figure 7).

**Figure 8:**
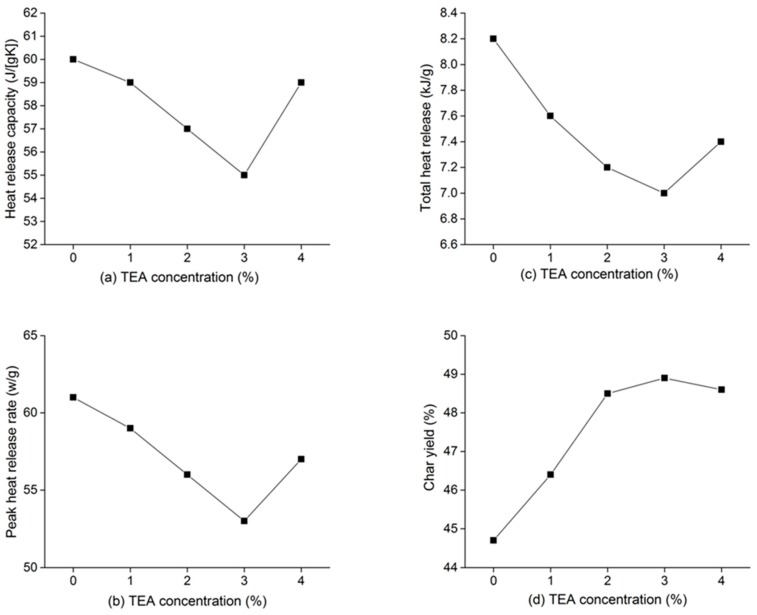
Heat release properties and char yield of the Nomex^®^/cotton fabric treated with 12% HFPO, 3% BTCA and TEA with different concentrations, cured at 170 °C for 3 min and subjected to one regular wash followed by a second wash with 0.01% Ca(NO_3_)_2_ added. (**a**) Heat release capacity; (**b**) Peak heat release rate; (**c**) Total heat release; and (**d**) Char yield.

Presented in Figure 8a–d are the HRC, PHRR, THR and char yield of the Nomex^®^/cotton fabric treated with 12% HFPO, 3% BTCA and TEA at different concentrations, cured and subjected to the two washing procedures as described above. The PHRR of the treated fabric without TEA is 61 w/g. It decreases to 59 w/g with 1% TEA added for the treatment, and it decreases further to 53 w/g with 3% TEA. The HRC and THR vs. TEA concentration curves follow the same trend (Figure 8). The char yield of the fabric treated without TEA is 44.7%. It increases from 46.4% to the maximum of 48.9% when the TEA concentration is increased from 1% to 3%, respectively (Figure 8). This phenomenon is consistent with the data presented in Table 6. The phosphorus concentration of the fabric treated with 12% HFPO and 3% BTCA and subjected two washing cycles reaches its maximum when TEA concentration is 3%. The data presented here clearly demonstrate the effectiveness of TEA for reducing PHRR and increasing the char formation for the thermal degradation of the Nomex^®^/cotton blend fabric treated with HFPO/BTCA/TEA.

Presented in Table 7 are the LOI and vertical burning char length of the Nomex^®^/cotton fabric treated with 12% HFPO, 3% BTCA and TEA at different concentrations, cured at 170 °C for 3 min and subjected to one regular washing cycle followed by a second wash with 0.01% Ca(NO_3_)_2_ added. The fabric treated using 3% TEA has the highest LOI (31.6%), which is two units higher than that of the fabric treated without TEA (29.6%). The char length of the fabric treated using TEA (36–39 mm) is also significantly lower than that of the fabric treated without TEA (45 mm). The data provide convincing evidence to prove the effectiveness of TEA for improving the fire performance of the HFPO/BTCA system on the blend fabric.

In our previous research [21], the same Nomex^®^/cotton fabric was treated with 24% HFPO, 8% BTCA, 2.5% H_3_PO_2_ and TEA with concentration ranging from 1% to 8%. Without laundering, all the treated Nomex/cotton fabric samples have the same concentrations of HFPO and H_3_PO_2_, both contain phosphorus, thus having the same phosphorus concentration, but they contain different TEA concentrations. The LOI (%) of the blend fabric increases from 37.2 to 40.6 as the TEA concentration increases from 0% to 8% [21]. Thus, the LOI data demonstrate that the use of TEA serves another function. TEA improves flame retardant performance of the treated Nomex^®^/cotton fabric by providing synergistic nitrogen.

### 3.4. The Laundering Durability of the Nomex^®^/Cotton Blend Fabric Treated with HFPO/BTCA/TEA

Presented in Table 8 are the weight gain, percent fixation, LOI and vertical burning char length of the blend fabric treated with 8% HFPO, 3% BTCA and 2% TEA, cured at 170 °C for 3 min and subjected to different number of home laundering washing cycles. The char length and LOI are 37 mm and 31.2%, respectively, after the treated fabric is subjected to one wash, indicating high flame retardant performance. The weight gain (5.2%) of the fabric after one wash is due to the bonding of HFPO/BTCA/TEA applied to the blend fabric. The percent fixation (92.3%) represents the percentage of the total HFPO/BTCA/TEA covalently bound to the fabric with respect to the prewash value. The treated fabric still has 87.2% fixation and the char length of the treated fabric becomes 66 mm after 25 washes. The flame retarding performance of the treated Nomex^®^/cotton blend fabric was well above the government regulation requirement in China where the fabric was produced and used. Chinese regulation (“GB 8965-1998: National Standard for Flame Retardant Protective Clothing”) requires flame retardant protective clothing to maintain vertical burning fabric char length ≤150 mm after 12 home laundering washes [30].

The treated blend fabric failed the vertical burning test after 50 washing cycles. We notice that 82.0% of the flame retardant system was still bound to the fabric and LOI reduces modestly from 27.0% to 26.2% as the number of washing cycle increases from 25 to 50. The treated fabric failed the vertical flammability test after 50 laundering cycles because the initial HFPO concentration used for the treatment is small (8%). The treated fabric could easily pass the vertical burning test after 50 washes if the initial HFPO concentration had been increased modestly.

## 4. Conclusions

1. Esterification of cotton by BTCA on the Nomex^®^/cotton blend fabric reduces PHRR of cotton. The treated blend fabric has marginally increased LOI and fails the fabric vertical burning flammability test.

2. When the Nomex^®^/cotton blend fabric is treated by the combination of HFPO and BTCA, HFPO was bound to cotton via BTCA’s esterification with both HFPO and cotton. The PHRR, HRC and *T*_PHRR_ were drastically reduced and the char yield was significantly increased due to the bonding of HFPO on cotton. The increase of fabric stiffness is an indication of the formation of HFPO/BTCA crosslinked polymeric network on the treated fabric. The treated fabric passed the fabric vertical burning flammability test. The char length of the vertical burning test reaches 30–50 mm at HFPO concentration of 6%–14%.

3. The free carboxy groups on the Nomex^®^/cotton blend fabric treated with HFPO/BTCA form insoluble calcium salts when the fabric was laundered. When TEA is used as a co-reactant, esterification between the free carboxy of BTCA and the hydroxy of TEA takes place, thus reducing the calcium deposition during multiple launderings. TEA also reacts with BTCA and becomes a part of the polymeric crosslinked network, thus improving the hydrolysis resistance of HFPO on the fabric. Inclusion of TEA in the HFPO/BTCA system definitively increases the flame resistant performance of treated blend fabric as indicated by shorter char length and higher LOI. The PHRR, HRC and THR are reduced, whereas char yield is increased.

4. The HFPO/BTCA/TEA system is able to impart high levels of flame resistance to the Nomex^®^/cotton blend fabric to achieve a short char length (~40 mm) at a low HFPO concentrations (~8%). It is a formaldehyde-free, odor-free and durable flame retardant system for the 65/35 Nomex^®^/cotton blend fabric.

## Figures and Tables

**Table 1:** The heat release properties and char yield of the Nomex^®^/cotton blend fabric treated with BTCA at different concentrations, cured at 170 °C for 3 min and finally subjected to one home laundering cycle.

BTCA (%)	HRC (J/(gK))	PHRR (w/g)	Char yield (%)
0.5	73	70	31.9
1	72	69	31.5
2	70	67	31.8
3	71	67	31.4
4	71	69	31.3
untreated	91	89	31.2

**Table 2:** The limiting oxygen index (LOI) and vertical burning flammability of the Nomex^®^/cotton blend fabric treated with BTCA, cured at 170 °C for 3 min and finally subjected to one home laundering.

BTCA (%)	LOI (%)	Char length (mm)
0.5	23.6	>300
1	23.5	>300
2	23.5	>300
3	23.7	>300
4	23.4	>300
untreated	22.8	>300

**Table 3:** The phosphorus and calcium concentrations of the Nomex^®^/cotton blend fabric treated with 3% BTCA and HFPO at different concentration and cured at 170 °C for 3 min.

HFPO (%)	Phosphorus (%)		Calcium (%) After 1 wash
	Before wash	After 1 wash	
6	0.46	0.37	0.075
8	0.65	0.49	0.073
10	0.75	0.51	0.071
12	0.94	0.60	0.067
14	1.03	0.64	0.065

**Table 4:** The heat release properties and char yield of the Nomex^®^/cotton blend fabric treated with 3% BTCA and HFPO at different concentration, cured at 170 °C for 3 min and finally subjected to one home laundering cycle.

HFPO (%)	HRC (J/(gK))	PHRR (w/g)	THR (kJ/g)	*T*_PHRR_ (°C)	Char yield (%)
6	58	57	8.7	314	41.9
8	56	55	8.4	307	42.5
10	53	52	7.8	308	44.1
12	52	51	7.5	304	46.5
14	51	50	7.0	298	48.0
untreated	91	90	9.2	371	31.2

**Table 5:** The LOI and vertical burning flammability of the Nomex^®^/cotton blend fabric treated with 3% BTCA and HFPO at different concentrations, cured at 170 °C for 3 min and finally subjected to one home laundering cycle.

HFPO (%)	LOI (%)	Char length (mm)
6	27.6	51
8	29.7	38
10	30.0	40
12	30.9	35
14	31.1	34
untreated	22.8	>300

**Table 6:** The phosphorus concentration of the Nomex^®^/cotton blend fabric treated with 12% HFPO, 3% BTCA and TEA with different concentration and cured at 170 °C for 3 min.

TEA (%)	Before wash (*P*_0_)	After 1 wash (*P*_1_)	After 2 washes (*P*_2_)	Ratio (*P*_2_/*P*_1_)
0	0.90	0.61	0.45	0.74
1	0.91	0.66	0.58	0.89
2	0.93	0.72	0.65	0.90
3	0.91	0.70	0.69	0.99
4	0.91	0.68	0.67	0.99

**Table 7:** LOI and vertical burning flammability of the Nomex^®^/cotton blend fabric treated with 12% HFPO, 3% BTCA and TEA with different concentration, cured at 170 °C for 3 min, and subjected to one regular wash followed by a second wash with 0.01% Ca(NO_3_)_2_ added.

TEA (%)	LOI (%)	Char length (mm)
0	29.6	45
1	30.9	37
2	31.2	36
3	31.6	39
4	31.4	37
untreated	22.8	>300

**Table 8:** The weight gain, fixation, LOI and vertical burning flammability of the Nomex^®^/cotton blend fabric treated with 8% HFPO, 3% BTCA and 2% TEA, cured at 170 °C for 3 min and finally subjected to different number of laundering cycles.

No. of laundering cycles	Weight gain (%)	Fixation (%)	LOI (%)	Char length (mm)
1	5.2	92.3	31.2	37
10	5.0	91.5	28.2	45
25	4.9	87.2	27.0	66
50	4.6	82.0	26.2	>300
Control	-	-	22.8	>300
